# Invasive Pulmonary Aspergillosis Diagnosis *via* Peripheral Blood Metagenomic Next-Generation Sequencing

**DOI:** 10.3389/fmed.2022.751617

**Published:** 2022-03-24

**Authors:** Xiaoxu Ma, Suping Zhang, Haizhou Xing, Huiling Li, Jiajun Chen, Haijun Li, Mengfan Jiao, Qingmiao Shi, Aiguo Xu, Lihua Xing, Weijie Cao

**Affiliations:** ^1^Department of Respiration, The First Affiliated Hospital of Zhengzhou University, Zhengzhou, China; ^2^Department of Hematology, The First Affiliated Hospital of Zhengzhou University, Zhengzhou, China; ^3^College of Public Health, Zhengzhou University, Zhengzhou, China; ^4^Department of Clinical Laboratory, The First Affiliated Hospital of Zhengzhou University, Zhengzhou, China; ^5^Department of Infectious Diseases, The First Affiliated Hospital of Zhengzhou University, Zhengzhou, China; ^6^Gene Hospital of Henan , Precision Medicine Center, The First Affiliated Hospital of Zhengzhou University, Zhengzhou, China

**Keywords:** invasive pulmonary aspergillosis, diagnosis, peripheral blood, metagenomic next generation sequencing, neutropenia

## Abstract

Invasive pulmonary aspergillosis (IPA) is one of the major causes of morbidity and mortality in immunocompromised patients such as hematological malignancies, hematopoietic stem cell transplantation, and solid organ transplantation. The diagnosis of IPA in these patients is still difficult because it has no obvious specificity in clinical symptoms, signs and imaging, and test sensitivity of blood 1,3-β-d-glucan test, galactomannan are low. Therefore, we still need to explore more diagnostic methods. In our study, *via* peripheral blood metagenomic next-generation sequencing (mNGS), five patients were tested positive for *Aspergillus* DNA and then quickly diagnosed as IPA. Out of the 5 cases, 1 was proven and 4 were probable IPA. The underlying diseases of the 5 patients were myelodysplastic syndrome (2 cases), acute myeloid leukemia (2 cases), and renal transplantation (1 case). Then they were diagnosed as IPA using other methods such as lung histopathology, bronchoalveolar lavage fluid (BALF) mNGS, and sputum culture or sputum mNGS. In case 1, sputum culture suggested *Aspergillus flavus*. In case 2, both Grocott methenamine silver (GMS) stain of lung histopathology and lung tissue mNGS suggested *Aspergillus* infection. In cases 3 and 4, BALF-mNGS suggested *Aspergillus* infection. In case 5, sputum mNGS suggested *Aspergillus* infection. In conclusion, detecting the cfDNA of *Aspergillus via* peripheral blood mNGS can be used to diagnose IPA and is a rapid and non-invasive diagnosis method.

## Introduction

Invasive pulmonary aspergillosis (IPA) is the most serious type of *Aspergillus*-related infection with the worst prognosis (1, 2). Risk factors for IPA include neutropenia, hematopoietic stem cell transplantation (HSCT), solid organ transplantation (SOT), and long-term use of corticosteroids (3, 4). A study on autopsy results showed that the incidence of IPA is increasing, and in spite of the improvement of diagnostic techniques, 75% of invasive fungal diseases still cannot be diagnosed before death (5). The mortality rate of IPA is high. The 90-day mortality rate of hematology patients combined with IPA who received and did not receive voriconazole prophylaxis was 46 and 59.3%, respectively (6). Delayed diagnosis and treatment were important causes of the high mortality of IPA (7).

At present, the commonly used clinical methods to diagnose IPA include chest computed tomography (CT), galactomannan test (GM test), direct microscopic examination, fungal culture, plasma, serum, whole blood or bronchoalveolar lavage fluid (BALF) polymerase chain reaction (PCR), and histopathological evaluation. The chest CT images of IPA are often atypical, and it is difficult to distinguish it from other infections using imaging. The 1,3-β-d-glucan test (G test), GM test, direct examination, fungal culture, and other tests also have disadvantages such as low sensitivity and poor specificity (8, 9). Although histopathology is the gold standard, patients with hematological diseases, HSCT, and SOT are often in poor physical condition and blood coagulation function, making invasive procedures to obtain tissue specimen often difficult to tolerate. The sensitivity and specificity of the GM test of BALF are higher than those of blood GM test and culture (8), but it can be easily affected by the patient's disease state, which makes its clinical application quite limited. Therefore, there is an urgent need for a rapid and non-invasive method to accurately diagnose IPA.

Metagenomic next-generation sequencing (mNGS) is increasingly used in the clinical diagnosis of infectious diseases. As a new technology that does not require culture, it can directly identify non-cultivable, fastidious, and non-bacterial (viral and fungal) pathogens from blood samples, with a higher sensitivity than conventional culture methods (10). Although it is difficult for *Aspergillus* to grow in peripheral blood, hyphae growing in the alveoli can penetrate the air and blood barrier, erode capillary endothelial cells, and invade small arteries and lung parenchyma. In that process, DNA fragments may enter the blood, so the PCR detection of peripheral blood can be used to diagnose IPA. PCR detection improves the possibility of early diagnosis of *Aspergillus*; however, the limitation of primer design and incomplete coverage may cause false negative results and delay the diagnosis. It has been confirmed that plasma mNGS can identify circulating DNA of respiratory pathogens in critically ill patients with bacterial pneumonia (11), but it is still unclear whether plasma mNGS can be used to diagnose IPA.

In this study, IPA was identified *via* peripheral blood mNGS, demonstrating its important role in the clinical diagnosis of IPA. Now we are reporting demographic information, clinical symptoms, diagnosis and treatment, and prognosis of the 5 patients. To our knowledge, this is the first report of IPA diagnosis *via* peripheral blood mNGS in China.

## Materials and Methods

We collected 5 patients whose peripheral blood mNGS test was positive for *Aspergillus* between September 9, 2020, and December 31, 2020, and recorded the demographic information, clinical symptoms, imaging examination results, laboratory examination results, treatment medication, and prognosis. We categorized IPA as proven, probable, and possible according to the 2019 European Organization for Research and Treatment of Cancer and the Mycoses Study Group Education and Research Consortium (EORTC/MSGERC) criteria (12). The galactomannan (GM) test was performed at least 3 times in each case, and we recorded the highest detection value. The positive threshold of GM test is 1.0 μg/L (12). This study was approved by the Ethics Committee of the First Affiliated Hospital of Zhengzhou University. The ethics approval number is 2021-KY-0439-002.

### Peripheral Blood mNGS

Peripheral venous blood specimens were collected using blood collection tube (BCT) tubes, centrifuged at 1,600 g for 10 min, and the plasma supernatant was used for cell-free DNA (cfDNA) extraction. Experiment quality was controlled by internal controls (IC) and external controls. To ensure that there were no mistakes in specimen tracking, 24 different ICs were designed. Each mNGS assay was run along with an external negative batch control consisting of 20 ng human cell line DNA (ATCC, CCL-243) in 600 μl 1M Tris-HCl buffer. DNA from all specimens, including the negative controls, was extracted using the TIANamp Micro DNA Kit (TIANGEN) and quantified using fluorometry (ThermoFisher Scientific). DNA libraries were constructed using an NGS library construction kit (Enzymatics) with unit dual index adapters. Libraries were pooled to be sequenced on Illumina NextSeq sequencers using a 75-cycle single-end sequencing strategy.

The primary sequencing output was demultiplexed with bcl2fastq version 2.20.0.422 with default parameters. Reads for quality trimming, low-complexity sequences, and adapter removal were performed using fastp version 0.19.5 (13). Specimens with fewer than 10 million reads after quality control (QC) were excluded in this study. The QC information of specimens is shown in online Supplementary Table 1. Reads mapped to human reference assembly GRCh38 were removed using bowtie2 version 2.3.4.3 (14). A set of criteria similar to the National Center for Biotechnology Information (NCBI) criteria was used for selecting representative genomes for microorganisms from the NCBI Nucleotide and Genome databases. The final database consisted of about 12,000 genomes. All putative microbial reads were aligned to our microorganism database with SNAP version 1.0beta.18 (15). The laboratory of our hospital adopts the mNGS process of Professor Wang Hui of Peking University People's Hospital (16).

## Results

**Figure 1:**
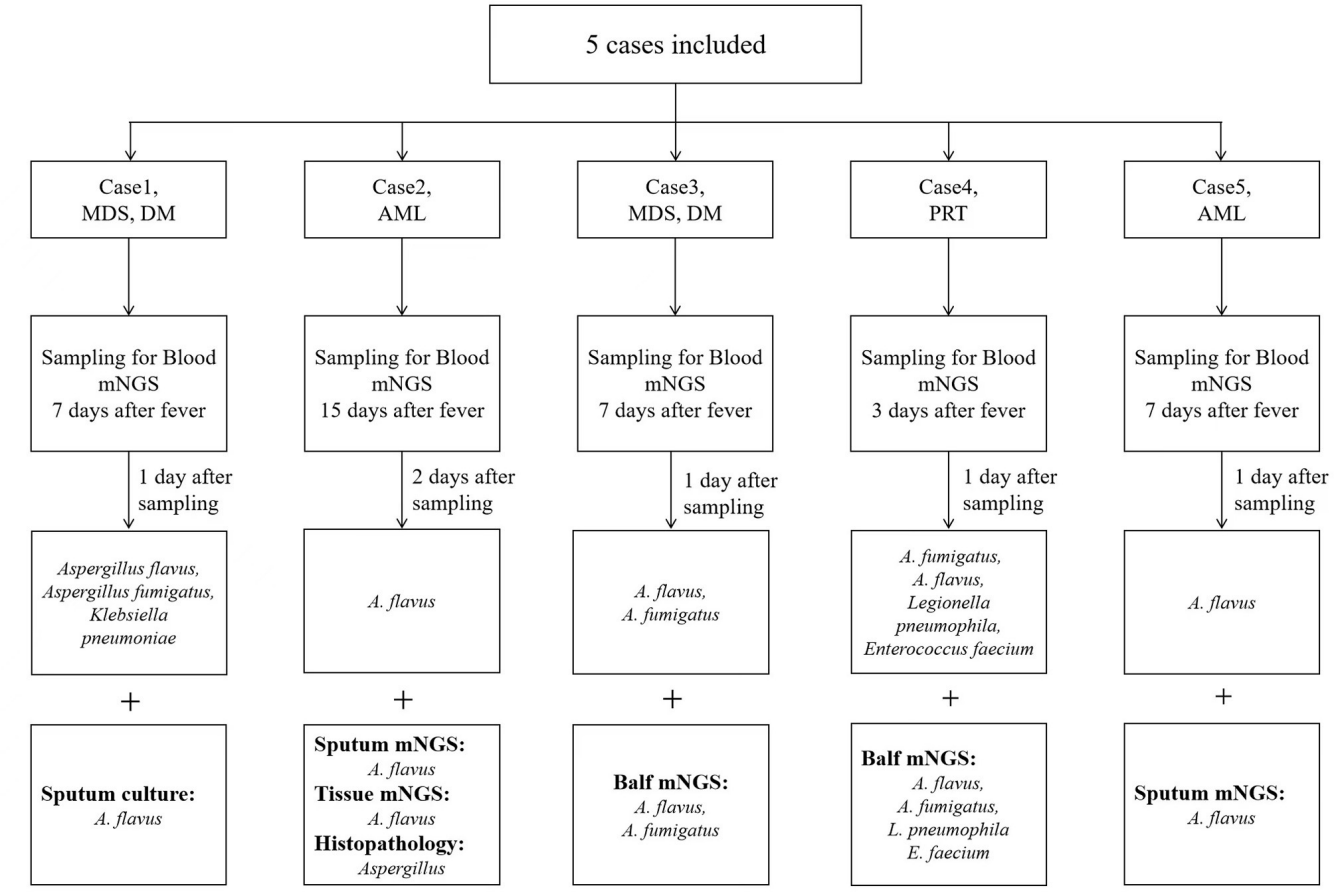
The diagnostic process of 5 cases.

We identified 5 patients who tested positive for *Aspergillus via* peripheral blood mNGS. Pathological or other pathogenic results confirmed that all the 5 patients had IPA. The flow diagram of included patients is shown in Figure 1. Demographic and clinical characteristics are shown in Table 1. The detailed medical records of those 5 patients are as follows.

**Table 1:** Demographic and clinical characteristics.

	**Case 1**	**Case 2**	**Case 3**	**Case 4**	**Case 5**
Age	55	21	56	55	39
Gender	Male	Female	Female	Female	Male
Underlying conditions	MDS, DM	AML	MDS, DM	PRT	AML
Fever	Yes	Yes	Yes	Yes	Yes
Cough	Yes	Yes	NA	Yes	NA
Sputum	White sticky sputum	Thin sputum	NA	Blood-stained sputum	NA
Chest pain	NA	Yes	NA	NA	NA
Neutropenia	Yes	Yes	Yes	Yes	Yes
Prognosis	Death	Improved	Death	Death	Improved

*MDS, myelodysplastic syndrome; DM, diabetes mellitus; NA, not available; AML, acute myeloid leukemia; PRT, post renal transplantation*.

### Case Descriptions

**Figure 2:**
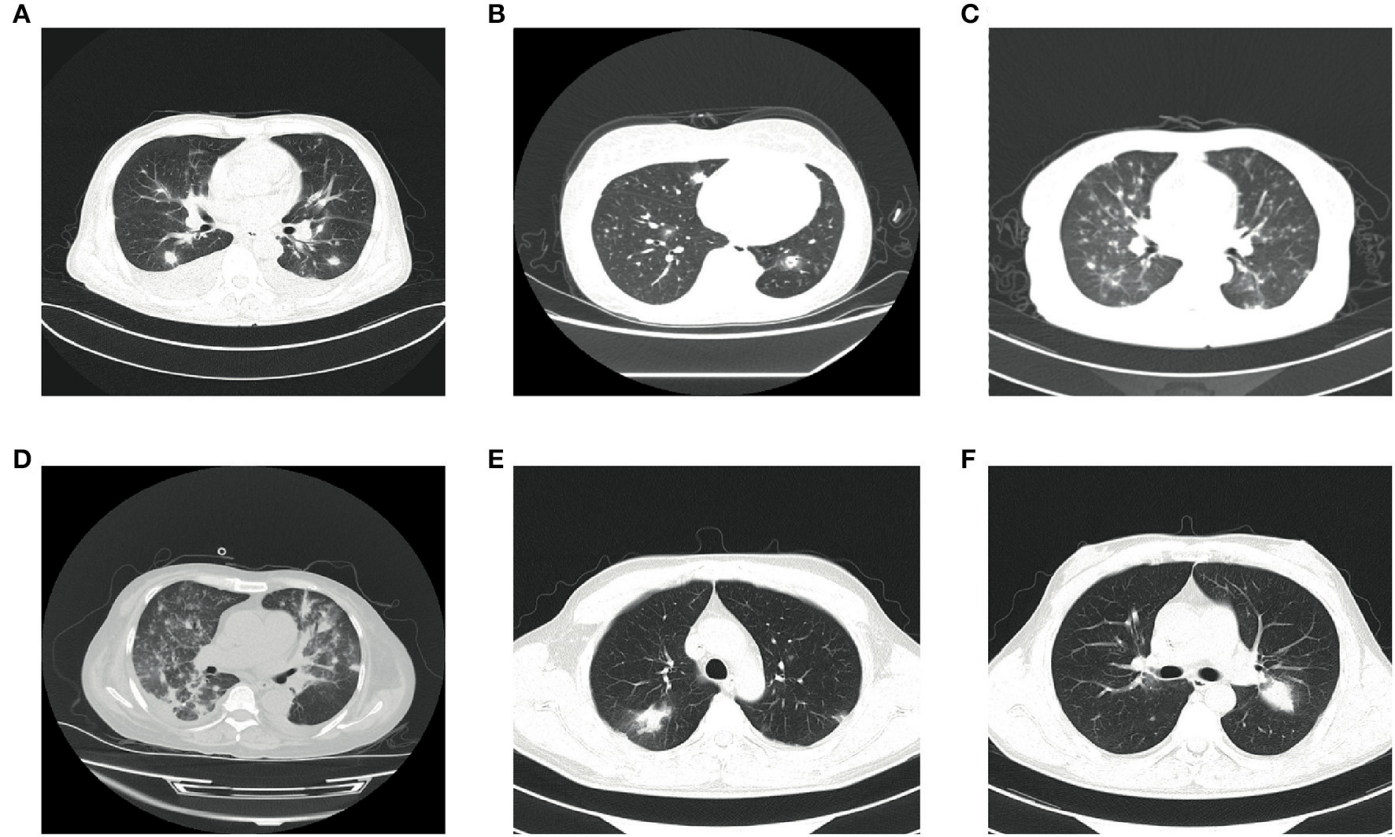
Findings of chest CT scan. **(A)** Chest CT of case 1 showing multiple small nodules in both lungs and a little pleural effusion on both sides. **(B)** Chest CT of case 2 showing multiple small nodules in both lungs and halo signs around some nodules. **(C)** Chest CT of case 3 showing multiple small nodules along the vascular bundles in both lungs and a little pleural effusion on both sides. **(D)** Chest CT of case 4 showing multiple irregular nodules in both lungs and a little pleural effusion on the right side. **(E,F)** Chest CT of case 5 showing multiple nodules and masses in both lungs.

**Case 1**, a male patient aged 55 years, was admitted to the hospital on September 11, 2020, with “fever and cough for 3 days.” The patient was diagnosed with myelodysplastic syndrome (MDS) in July 2019. With type II diabetes, the patient's serum glucose was controlled well. On August 20, 2020, “decitabine + CAG (cytarabine + acramomycin + granulocyte colony-stimulating factor) chemotherapy regimen” was performed, and there was mild bone marrow suppression after chemotherapy. On September 8, 2020, the patient developed fever, with a maximum body temperature of 39.6°C, accompanied by cough, white sticky sputum, chest tightness, and asthma. Laboratory examinations on admission are as follows (Table 2): white blood cell (WBC) count, 0.32 × 10^9^ cells/L; red blood cell (RBC) count, 2.41 × 10^12^ cells/L; hemoglobin (Hb), 79.3 g/L; platelet (PLT), 6 × 10^9^ cells/L; neutrophile (NE), 0.03 × 10^9^ cells/L; lymphocyte (Ly), 0.28 × 10^9^ cells/L; procalcitonin (PCT), 3.45 ng/ml; C-reactive protein (CRP), 131 mg/L; G test, <10 pg/ml; GM test, <0.25 μg/L; and chest CT on September 14, 2020: multiple small nodules could be seen in both lungs (Figure 2A). After admission, blood culture and sputum culture were conducted, and the patient was given anti-infective treatment with biapenem, voriconazole, and teicoplanin, but body temperature did not improve significantly. On September 15, 2020, peripheral blood was collected for mNGS. On September 16, 2020, the results of peripheral blood mNGS suggested *Aspergillus flavus* and *Aspergillus fumigatus* infection (Table 3). On September 18, 2020, sputum culture suggested *A. flavus* infection. After continued voriconazole treatment for 1 week, the patient died of secondary mucor infection.

**Table 2:** Laboratory examination and treatment.

	**Case 1**	**Case 2**	**Case 3**	**Case 4**	**Case 5**
Laboratory examination				
WBC (10∧9/L)	0.32	0.02	0.4	0.21	0.22
RBC (10∧12/L)	2.41	3	3.4	2.97	1.7
Lymphocyte (10∧^9^/L)	0.28	0.02	0.34	0.06	0.14
Monocyte (10∧^9^/L)	0.01	0.08	0	0	0.01
Neutrophile (10∧9/L)	0.03	0.17	0.03	0.15	0.06
Platelet (10∧9/L)	6	26	267	22	11
Hemoglobin (g/L)	79.3	95	96.5	86	57.1
Albumin (g/L)	29.1	32.7	21.9	29.2	32.1
LDH (U/L)	234	86	73	840	88
CRP(mg/L)	131	108	143	234.3	99.4
PCT (ng/mL)	3.45	0.747	6.07	13.4	68.3
G test	Negative	Negative	Positive	Negative	Negative
GM test	Negative	Negative	Positive	Negative	Negative
Methods of diagnosis				
Histopathology	NA	*Aspergillus*	NA	NA	NA
Blood mNGS	*Aspergillus flavus, Aspergillus fumigatus*	*A. flavus*	*A. flavus, A. fumigatus*	*A. fumigatus, A. flavus*	*A. flavus*
Blood culture	Negative	Negative	Negative	Negative	Negative
Sputum culture	*A. flavus*	Negative	Negative	Negative	Negative
BALF culture	NA	Negative	Negative	Negative	NA
BALF mNGS	NA	NA	*A. flavus, A. fumigatus*	*Legionella pneumophila, Enterococcus faecium*, *A. fumigatus*, *A. flavus*	NA
Sputum mNGS	NA	*A. flavus*	NA	NA	*A. flavus*
Tissue mNGS	NA	*A. flavus*	NA	NA	NA
Treatment	Voriconazole	Posaconazole Voriconazole, AMB, LMB	Voriconazole, Caspofungin	Voriconazole, Caspofungin	Posaconazole

*WBC, white blood cell; RBC, red blood cell; LDH, lactic dehydrogenase; CRP, C-reactive protein; PCT, procalcitonin; G test, 1,3-β-D-glucan test; GM test, galactomannan test; mNGS, metagenomic next-generation sequencing; BALF, bronchoalveolar lavage fluid; NA, not available; AMB, amphotericin B; LMB, amphotericin B liposome*.

**Table 3:** Results of peripheral blood mNGS.

**Patient NO**.	**Sample**	**mNGS testing results**	**mNGS sequence number**
1	peripheral blood	*Aspergillus flavus*	2,067
		*Aspergillus fumigatus*	568
		*Klebsiella pneumoniae*	38
2	peripheral blood	*A. flavus*	9
3	peripheral blood	*A. flavus*	502
		*A. fumigatus*	1
4	peripheral blood	*A. fumigatus*	2,680
		*A. flavus*	2
		*Legionella pneumophila*	174
		*Enterococcus faecium*	77
5	peripheral blood	*A. flavus*	44

*mNGS, metagenomic next-generation sequencing*.

**Case 2**, a female patient aged 21 years, was admitted to the hospital on November 26, 2020, with “acute myeloid leukemia (AML) diagnosed for more than 11 months and fever for 6 days.” On November 5, 2020, the patient received “decitabine 10 mg × 10 d” chemotherapy, severe bone marrow transplantation after chemotherapy, and oral posaconazole to prevent fungal infection. On September 20, 2020, the patient developed fever, with a maximum body temperature of 39°C, accompanied by cough and chest pain. Laboratory examinations on admission are as follows (Table 2): WBC count, 0.02 × 10^9^ cells/L; RBC count, 3 × 10^12^ cells/L; Hb, 95 g/L; PLT, 26 × 10^9^ cells/L; NE, 0.17 × 10^9^ cells/L; Ly, 0.02 × 10^9^ cells/L; PCT, 0.747 ng/ml; CRP, 108 mg/L; G test, <10 pg/ml; and GM test, <0.25 μg/L. Under treatment with cefoperazone and sulbactam + levofloxacin + voriconazole anti-infection, fever did not improve. Chest CT scan on December 3, 2020, showed multiple nodules in both lungs and halo signs around the nodules (Figure 2B). On December 5, 2020, peripheral blood was collected for mNGS. On December 7, 2020, the results of peripheral blood mNGS suggested *A. flavus* infection, combined with intravenous infusion of amphotericin B liposome and aerosol inhalation of amphotericin B, then body temperature returned to normal 12 days later. Reexamination of chest CT on January 3, 2021, showed no obvious absorption of lung nodules. At this time, the patient's blood platelets reached 85 × 10^9^ cells/L. To further confirm the diagnosis, CT-guided percutaneous lung puncture was conducted for pathology and mNGS. Grocott methenamine silver (GMS) stain of lung histopathology revealed septal hyphae with 45 degree branches. and lung tissue mNGS suggested *A. flavus* infection (Table 3). The patient is still being treated with amphotericin B antifungal therapy.

**Case 3**, a female patient aged 56 years, was admitted to the Department of Endocrinology on August 3, 2020, with “thirsty, excessive drinking for more than 6 years and sore throat for 4 days.” Laboratory examinations on admission are as follows (Table 2): WBC count, 0.4 × 10^9^ cells/L; RBC count, 3.4 × 10^12^ cells/L; Hb, 96.5 g/L; PLT, 267 × 10^9^ cells/L; NE, 0.03 × 10^9^ cells/L; Ly, 0.34 × 10^9^ cells/L; PCT, 6.07 ng/ml; CRP, 143 mg/L; G test, 333.56 pg/ml; and GM test, 1.01 μg/L. Chest CT on admission was normal. The patient was diagnosed with type II diabetes and hyperthyroidism. Later, to further identify the cause of agranulocytosis, the patient was transferred to the Department of Hematology for further treatment. After the transfer, the patient was diagnosed as MDS using bone marrow aspiration, bone marrow biopsy, and flow cytometry. During the diagnosis, the patient developed a high fever and had a negative blood culture. On September 6, 2020, reexamination of chest CT revealed multiple small nodules in both lungs (Figure 2C). She was treated with voriconazole first, but she still had high fever. On September 8, 2020, peripheral blood was collected for mNGS. On September 9, 2020, the results of peripheral blood mNGS suggested *A. flavus* and *A. fumigatus* infection (Table 3). Although voriconazole has been used for the treatment of *Aspergillus*, the patient still had high fever. To further identify the pathogen, on September 12, 2020, a fiberoptic bronchoscope was made for her, and the BALF was taken for bacterial culture, fungal culture, immunofluorescence staining, and mNGS. BALF-mNGS suggested *A. flavus* and *A. fumigatus* infection, but bacterial culture, fungal culture, and immunofluorescence staining were negative. The patient gave up medical treatment and died on September 16, 2020.

**Case 4**, a female patient aged 55 years, was admitted to the hospital on November 4, 2020, with “abnormal renal function for 3 years and regular dialysis for 3 years.” On November 5, 2020, the patient was given an allograft renal transplantation under general anesthesia. After operation, intravenous injection of Cefminox, ganciclovir, caspofungin, ganciclovir, and oral sulfonamide were used to prevent infection and oral tacrolimus and mycophenolate mofetil to prevent graft-vs.-host disease. Fever developed on the 20th day after the operation, and the highest body temperature was 39°C. Laboratory examinations on admission are as follows (Table 2): WBC count, 0.21 × 10^9^ cells/L; RBC count, 2.97 × 10^12^ cells/L; Hb, 86 g/L; PLT, 22 × 10^9^ cells/L; NE, 0.15 × 10^9^ cells/L; Ly, 0.06 × 10^9^ cells/L; PCT, 13.4 ng/ml; CRP, 234.3 mg/L; G test, <10 pg/ml; and GM test, <0.25 μg/L. Chest CT showed multiple irregular nodules in both lungs and a little pleural effusion on the right side (Figure 2D). With switching to meropenem, voriconazole, and doxycycline for anti-infective therapy, body temperature did not improve significantly. On November 28, 2020, blood and BALF were collected for mNGS. The test results both suggested *Legionella pneumophila, Enterococcus faecium, A. fumigatus*, and *A. flavus* infection (Table 3). On December 6, 2020, medical treatment failed and the patient died.

**Case 5**, a male patient aged 39 years, was admitted to the hospital with a diagnosis of AML for more than 6 months and fever for 5 h. On October 12, 2020, the patient was given “decitabine + CAG” chemotherapy for 14 days. After chemotherapy, the patient suffered severe bone marrow suppression. On October 30, 2020, the patient developed a high fever, with a body temperature of 39.7°C, and the body temperature could not return to normal on its own. Laboratory examinations on admission are as follows (Table 2): WBC count, 0.22 × 10^9^ cells/L; RBC count, 1.7 × 10^12^ cells/L; Hb, 57.1 g/L; PLT, 11 × 10^9^ cells/L; NE, 0.06 × 10^9^ cells/L; Ly, 0.14 × 10^9^ cells/L; PCT, 68.3 ng/ml; CRP, 99.4 mg/L; G test, <10 pg/ml; and GM test, <0.25 μg/L. The fever was still high after treatment with imipenem and cilastatin, teicoplanin, posaconazole suspension. On November 3, 2020, chest CT scan showed multiple nodules and masses in both lungs (Figures 2E,F). On November 7, 2020, blood and sputum mNGS both suggested *A. flavus* (Table 3). The anti-infective treatment plan was adjusted to cefoperazone and sulbactam + tigecycline antibacterial treatment, and the plasma concentration of posaconazole was 2.21 μg/ml. The body temperature returned to normal on November 18, 2020. The patient is still taking oral posaconazole suspension and continued regular chemotherapy since December 10, 2020, to treat AML. The patient is generally in good condition and is still being followed up.

## Discussion

In recent years, the incidence of invasive aspergillosis (IA) has increased rapidly. The estimated number of patients worldwide has risen from 200,000 to more than 300,000 cases per year. The average annual incidence is 4.1 cases per 100,000, of which IPA accounts for more than 90%. The mortality rate is still very high (17). Currently, the diagnosis of IPA is difficult, and early and accurate diagnosis is one of the key steps to effectively treat infections and reduce high mortality (18). Non-specific clinical and radiological manifestations and routine diagnostic methods may delay the correct diagnosis. Of the 5 patients included in this study, 2 had MDS as the underlying disease, 2 had AML, and 1 had renal transplantation, and all of them were at high risk of IPA. After treatment, 3 patients died, giving a mortality rate of 60%. The clinical symptoms of those 5 patients were fever and cough. PCT and CRP were higher than normal, which could not be distinguished from bacterial infections by clinical symptoms and laboratory tests. The chest imaging showed multiple nodules in both lungs, and only 1 case had a halo sign, lacking the typical imaging findings of IPA. Although the 5 patients were all in high-risk groups of IPA, none of them met the diagnostic criteria of EORTC/MSGERC IPA before mNGS. In our study, peripheral blood mNGS rapidly diagnosed *Aspergillus* infection, and then combined with the patient's chest CT, the pulmonary infection was considered as IPA.

These 5 patients were not only diagnosed with *Aspergillus* infection *via* peripheral blood mNGS, but 3 of them were also tested for combined bacterial infection, and blood cultures collected during their diagnosis and treatment were all negative. Previous studies had shown that the positive rate of peripheral blood culture in infectious diseases was only 1%, and the positive rate of peripheral blood mNGS could reach 24%, suggesting that peripheral blood mNGS has great diagnostic value in infectious diseases (19). Our study shows that peripheral blood mNGS can simultaneously detect bacterial and fungal infections that cause lung infections, which helps to rapidly clarify the condition and adjust the treatment plan.

The current methods for the diagnosis of IPA mainly include histopathology, direct examination method, fungal culture, fungal antigen and antibody detection, and molecular biology detection. All guidelines of *Aspergillus* diagnosis and treatment clearly point out that histopathology and sterile body fluid culture are the gold standards for the diagnosis of IPA. However, the proportion of proven IPA is extremely low because most patients in clinic cannot tolerate invasive procedures required by histopathology, and the positive rate of fungal culture is also extremely low. The G and GM tests are the two most used biomarkers of *Aspergillus*. But both the blood G and BALF-G tests have relatively low sensitivity and poor specificity for the diagnosis of IPA (20). The GM test, especially the BALF-GM test, has high sensitivity and specificity in patients with agranulocytosis and IPA and is currently the most widely used IA biomarker in clinical practice. However, the problem that puzzles clinicians is that the cutoff value of the BALF-GM test is still not uniform, and the sensitivity and specificity are different under different cutoff values (21). Infectious Diseases Society of America (IDSA) strongly recommends that patients with suspected IPA undergo bronchoscopy and obtain BALF for routine culture, cytology, and GM tests. However, in clinical practice, severely infected patients may be complicated by severe hypoxemia, bleeding, or thrombocytopenia, and invasive operations such as bronchoscopy will be difficult to implement, so relying on the BALF-GM test to diagnose IPA is not feasible (22). According to the diagnostic criteria of EORTC/MSGERC for IPA, we reported 5 patients, of whom 1 case was proven and 4 cases were probable. However, only 1 patient had a positive blood GM test, and 3 cases of BALF-GM test were all negative. Therefore, clinical attention should also be paid to the issue of false negative blood GM test and BALF-GM test.

Due to the high morbidity and mortality of IPA, a non-invasive detection method is urgently needed for the rapid and accurate diagnosis of IPA in clinical practice. It was found that DNA fragments of pathogens that can cause different infection sites in human body can be found in purified plasma. Sequencing the cfDNA of this microorganism can improve the possibility of non-invasive detection of multiple infections (17, 23, 24). PCR is recommended by the EORTC/MSGERC as a method for detecting *Aspergillus*, but research shows that the sensitivity and specificity of *Aspergillus* PCR analysis methods from different vendors are quite different. The sensitivity of Artus Aspergillus RG PCR assay was only 47.6%, while the sensitivity of MycAssay Aspergillus PCR was 61.9% (25). Another study showed that the sensitivity of GM and *Aspergillus* PCR in blood was even lower, only 31 and 0% (21). Due to the lack of uniform standards in different studies and environments and the huge differences in diagnostic performance, the exact role of PCR in the diagnosis of IPA remains controversial. mNGS is another molecular diagnostic technique used in the diagnosis of infectious diseases in recent years (26). Hong et al. used plasma NGS technology for the first time in 2018 to identify the pathogens of confirmed invasive fungal infections, including *Aspergillus* and non-*Aspergillus* species. Among the 9 patients, 7 cases of plasma NGS detected the same fungus found in the biopsy at the genus level (24). Our research has also confirmed that peripheral blood mNGS can be used to diagnose IPA, but the detection efficiency of peripheral blood mNGS still needs to be confirmed by large-scale clinical trials. Peripheral blood specimens of the 5 patients in this study were all collected after the onset of fever. No more peripheral blood mNGS test was performed during the course of the disease. Therefore, it is still unknown when *Aspergillus* cfDNA would exist in the peripheral blood and when to collect peripheral blood. The application of IPA diagnosis *via* peripheral blood mNGS is a subject that needs to be further explored. Studies have confirmed that antifungal treatment can reduce the sensitivity of PCR detection of peripheral blood *Aspergillus* (27), so collecting peripheral blood for mNGS early in the course of the disease may increase the diagnostic rate of IPA.

Our study has some limitations. First, this is a retrospective study that may be biased. The 5 cases included in this study cannot tolerate CT guided percutaneous lung puncture or tracheal puncture under tracheoscopy due to low platelet or poor physical condition, and only case 2 had pathological findings for diagnosis. Although cases 1, 3, 4, and 5 had microbiological evidence, they were not the gold standard for the diagnosis of IPA after all, so there is a slight chance that they are not *Aspergillus* infection. Second, the subjects are all patients with neutropenia, and the results of the study may not be applicable to patients with non-neutropenia IPA. Third, the sample size is small. During the diagnosis and treatment of these patients, the peripheral blood mNGS test was performed only once. It is impossible to confirm the existence time of *Aspergillus* cfDNA in the peripheral blood. The time to collect peripheral blood for the mNGS test is not yet clear.

In conclusion, our study confirmed that detecting the cfDNA of *Aspergillus via* peripheral blood mNGS can be used to diagnose IPA and is a rapid and non-invasive diagnosis method.

## Data Availability Statement

The datasets presented in this study can be found in online repositories. The name of the repository and accession number can be found below: National Center for Biotechnology Information (NCBI) BioProject, https://www.ncbi.nlm.nih.gov/bioproject/, PRJEB43573.

## Ethics Statement

The studies involving human participants were reviewed and approved by the Ethics Committee of the First Affiliated Hospital of Zhengzhou University. Written informed consent for participation was not required for this study in accordance with the national legislation and the institutional requirements.

## Author Contributions

XM, WC, AX, and LX conceived the project. JC, MJ, QS, and HaL performed the experiments. SZ, WC, and HX collected cases. XM and HuL analyzed and interpreted patient data. XM wrote the manuscript. All authors have read and approved the final manuscript.

## Funding

This research was supported by the Beijing Medical and Health Public Welfare Foundation(YWJKJJHKYJJ-B182121-1).

## Conflict of Interest

The authors declare that the research was conducted in the absence of any commercial or financial relationships that could be construed as a potential conflict of interest.

## Publisher's Note

All claims expressed in this article are solely those of the authors and do not necessarily represent those of their affiliated organizations, or those of the publisher, the editors and the reviewers. Any product that may be evaluated in this article, or claim that may be made by its manufacturer, is not guaranteed or endorsed by the publisher.
